# Multi-drug use among patients with multiple sclerosis: A cross-sectional study of associations to clinicodemographic factors

**DOI:** 10.1038/s41598-019-40283-5

**Published:** 2019-03-06

**Authors:** Niklas Frahm, Michael Hecker, Uwe Klaus Zettl

**Affiliations:** Neuroimmunology Section, Department of Neurology, University of Rostock, Rostock, Germany

## Abstract

Multiple sclerosis (MS) is the most prevalent immune-mediated disease affecting the central nervous system. A treatment strategy with multiple therapies is a frequent clinical scenario. Unmonitored multi-drug use can lead to adverse outcomes, higher health care costs and medication non-adherence. The primary aim of this study was to evaluate the frequency of polypharmacy and related clinicodemographic factors in a single-center MS patient cohort. Furthermore, medication aspects of therapy management were examined. After the patients agreed to participate in the study, data were collected through patient interviews, patient records and clinical investigations. Subsequently, a statistical data analysis regarding various medication subgroups and polypharmacy (use of at least five drugs) was performed. Polypharmacy was observed in 56.5% of the patients (N = 306). High degrees of disability (odds ratio [OR] = 1.385), comorbidities (OR = 4.879) and inpatient treatment (OR = 5.146) were associated with a significantly higher risk of polypharmacy (*p* ≤ 0.001). Among patients with polypharmacy, disease-modifying drugs, antihypertensives, gastrointestinal drugs, thrombosis prophylactics, osteoporosis medications and sedatives were frequently used. In summary, polypharmacy plays a large role in MS patients, especially in those with higher degrees of disability, those with comorbidities and those treated in an inpatient setting.

## Introduction

With 2.3 million people affected globally^1^, multiple sclerosis (MS) is the most frequent immune-mediated disease of the central nervous system^2^. It causes pathological loss of synapses, demyelination and axonal damage, which can lead to various combinations of symptoms^3^. Both genetic and environmental factors play a role in the manifestation of the disease^4–6^. There is a risk of developing MS in any age group, while most patients are diagnosed between the ages of 20 and 49 years^7^.

The introduction of interferon-beta-1b preparations in 1993 as the first disease-modifying drugs (DMDs) heralded the development of a range of new immunomodulatory drugs for MS^8–11^. Meanwhile, 16 different DMDs have been approved^12^. Considering the course of disease, quite diverse symptoms can arise, such as sensory disturbances^13^, pareses, spasticity^14^, fatigue^15^, pain^16^, emotional disturbances^17^ and coordination disturbances^18^. Therefore, in addition to the DMDs, targeted symptomatic therapeutic approaches, treatment of comorbidities besides MS and individual use of complementary medicines play a substantial role for the patients’ quality of life^19^. Given such complex therapeutic scenarios, it is essential to take polypharmacy into account. In our study, polypharmacy is defined as the use of five or more medications, which constitutes the most common definition^20–25^.

In the USA, 10% of the general population and 30% of the older generation is taking five or more medications simultaneously^26–28^. Similar rates of polypharmacy have been observed in other countries, such as the United Kingdom^29^, China^30^ and India^31^. Older people are often affected by polypharmacy as they suffer more frequently from comorbidities and therefore take more medications. For instance, previous research has shown a correlation between polypharmacy and gut microbiota composition in aging, leading to a detrimental clinical outcome^32^. Possible consequences of an inappropriate monitoring of polypharmacy in affected patients include rising socioeconomic costs and side effects^33^, serious drug interactions^34^, lack of adherence due to medication complexity^35^ and rehospitalizations^36^. Until now, the number of studies addressing partial aspects of polypharmacy in MS patients is limited^37–40^.

For this reason, the primary aim of our study was to examine the frequency of polypharmacy in a single-center MS patient cohort. Furthermore, we analyzed the sociodemographic and clinical-neurological factors associated with polypharmacy as well as medication aspects of therapy management. Finally, we examined differences in medication between younger and older MS patients, between outpatients and inpatients and between patients with and without polypharmacy (PwP and Pw/oP).

## Results

### Study population

The sociodemographic data of the study population, comprising 306 patients with a diagnosis of MS (N = 300) or clinically isolated syndrome (CIS) (N = 6), are presented in Table 1. The average age was 48.7 years and 71.2% of the patients were women. Over half of the patients were retired due to their health situation or due to age, while 37.6% were still in employment. Approximately three quarters (72.5%) of the patients were in a partnership and almost the same amount (73.5%) had at least one child. Nearly half of the patients (48.0%) had one sibling, while 38.9% had two or more.

**Table 1:** Sociodemographic data of the examined MS patients (N = 306).

	N (%)	Range	M (SD)^a^ or median^b^
Gender			
Female	218 (71.2)		
Male	88 (28.8)		
Age (Years)		19–86	48.7 (13.1)^a^
≤29	23 (7.5)		
30–39	64 (20.9)		
40–49	64 (20.9)		
50–59	96 (31.4)		
≥60	59 (19.3)		
School years		6–16	10^b^
Educational level			
No training	6 (2.0)		
Skilled worker	206 (67.3)		
Technical college	19 (6.2)		
University	75 (24.5)		
Employment status			
In training	6 (2.0)		
Employed	115 (37.6)		
Unemployed	10 (3.3)		
Retiree	168 (54.9)		
Others	7 (2.3)		
Partnership			
Yes	222 (72.5)		
No	84 (27.5)		
Place of residence			
Rural community	85 (27.8)		
Provincial town	57 (18.6)		
Medium-sized town	43 (14.1)		
City	121 (39.5)		
Number of children		0–4	1^b^
0	81 (26.5)		
1	87 (28.4)		
≥2	138 (45.1)		
Number of siblings		0–13	1^b^
0	40 (13.1)		
1	147 (48.0)		
≥2	119 (38.9)		

M, mean value; MS, multiple sclerosis; N, number of patients; SD, standard deviation. ^a^Mean value (standard deviation); ^b^median.

The clinical-neurological data of the patient cohort are presented in Table 2. The median EDSS score was 3.5, with individual scores ranging between 1.0 and 9.0. The majority of the patients (62.7%) had CIS/relapsing-remitting MS (RRMS), followed by secondary progressive MS (SPMS) (26.1%) and primary progressive MS (PPMS) (11.1%). The disease duration varied between 6 weeks and 50 years, with a median of 11 years. Almost two thirds of the patients (64.4%) suffered from comorbidities. The proportion of inpatients (52.3%) was roughly equal to the proportion of outpatients (47.7%).

**Table 2:** Clinical data of the examined MS patients (N = 306).

	N (%)	Range	Median
EDSS		1.0–9.0	3.5
1.0–3.5	170 (55.5)		
4.0–5.5	47 (15.4)		
≥6.0	89 (29.1)		
Disease course			
CIS/RRMS	192 (62.7)		
SPMS	80 (26.1)		
PPMS	34 (11.1)		
Disease duration (Years)		0–50	11
0*−5	88 (28.8)		
6–10	57 (18.6)		
11–15	54 (17.6)		
16–20	52 (17.0)		
≥21	55 (18.0)		
Comorbidities			
Pw/oSI	109 (35.6)		
PwSI	197 (64.4)		
Patient Care			
Outpatients	146 (47.7)		
Inpatients	160 (52.3)		

CIS, clinically isolated syndrome; EDSS, expanded disability status scale; MS, multiple sclerosis; N, number of patients; PPMS, primary progressive MS; PwSI, patients with secondary illnesses; Pw/oSI, patients without secondary illnesses; RRMS, relapsing-remitting MS; SPMS, secondary progressive MS.

*Six weeks as the lowest disease duration.

### Comparison between patients with polypharmacy and patients without polypharmacy

In the analysis of the examined patients according to all medications taken, 56.5% were categorized as PwP. Conducting the analysis with regard to long-term medications only, the proportion of PwP amounted to 42.2%. In total, the average number of medications taken by the patients was 5.7, with a range from 1 to 19.

Studying polypharmacy according to all medications, PwP were, on average, significantly older than Pw/oP (53.0 vs. 43.0 years) and had significantly more children (1.4 vs. 1.2) (Table 3). Pw/oP had a significantly higher number of school years (10.9 vs. 10.4 years) and were more frequently employed (52.6% vs. 26.0%) than PwP. After adjusting the *p*-values by means of false discovery rate (FDR) correction, all of the aforementioned differences remained significant, except for the number of children. Defining polypharmacy by using long-term medications only, the same sociodemographic variables showed significant differences between PwP and Pw/oP and these differences also remained significant following FDR correction.

**Table 3:** Comparison of sociodemographic data between patients with and without polypharmacy.

N	Polypharmacy (all medications)			Polypharmacy (long-term medications only)		
	Pw/oP	PwP	Statistics	Pw/oP	PwP	Statistics
	133	173		177	129	
Gender^c^			*p* = 0.799^Fi^			*p* = 1.000^Fi^
Female	96 (72.2)	122 (70.5)		126 (71.2)	92 (71.3)	
Male	37 (27.8)	51 (29.5)		51 (28.8)	37 (28.7)	
Age (Years)^a^	43.0 (11.4)	53.0 (12.7)	**t**_**(304)**_** = −7.170**, ***p* < 0.001**^**t**^	43.2 (11.6)	56.2 (11.2)	**t**_**(304)**_** = −9.815**, ***p* < 0.001**^**t**^
School years^a^	10.9 (1.4)	10.4 (1.3)	**t**_**(277.487)**_** = 3.322**, ***p* = 0.001**^t^*	10.9 (1.4)	10.2 (1.2)	**t**_**(289.722)**_** = 4.599**, ***p* < 0.001**^t^*
Educational level^c^			X^2^_(3)_ = 4.634, *p* = 0.201^Chi^			X^2^_(3)_ = 3.301, *p* = 0.348^Chi^
No training	3 (2.3)	3 (1.7)		3 (1.7)	3 (2.3)	
Skilled worker	81 (60.9)	125 (72.3)		113 (63.8)	93 (72.1)	
Technical college	9 (6.8)	10 (5.8)		11 (6.2)	8 (6.2)	
University	40 (30.1)	35 (20.2)		50 (28.2)	25 (19.4)	
Employment status^c^			**X**^**2**^_**(5)**_** = 38.139**, ***p* < 0.001**^**Chi**^			**X**^**2**^_**(5)**_** = 47.944**, ***p* < 0.001**^**Chi**^
In training	5 (3.8)	1 (0.6)		6 (3.4)	0 (0.0)	
Employed	70 (52.6)	45 (26.0)		89 (50.3)	26 (20.2)	
Unemployed	6 (4.5)	4 (2.3)		9 (5.1)	1 (0.8)	
Retiree	47 (35.3)	121 (69.9)		68 (38.4)	100 (77.5)	
Other	5 (3.8)	2 (1.2)		5 (2.8)	2 (1.6)	
Partnership^c^			*p* = 0.301^Fi^			*p* = 0.245^Fi^
Yes	101 (75.9)	121 (69.9)		133 (75.1)	89 (69.0)	
No	32 (24.1)	52 (30.1)		44 (24.9)	40 (31.0)	
Place of residence^c^			X^2^_(3)_ = 1.307, *p* = 0.728^Chi^			X^2^_(3)_ = 3.081, *p* = 0.379^Chi^
Rural community	39 (29.3)	46 (26.6)		53 (29.9)	32 (24.8)	
Provincial town	23 (17.3)	34 (19.7)		30 (16.9)	27 (20.9)	
Medium-sized town	16 (12.0)	27 (15.6)		21 (11.9)	22 (17.1)	
City	55 (41.4)	66 (38.2)		73 (41.2)	48 (37.2)	
Number of children^a^	1.2 (1.0)	1.4 (1.0)	**t**_**(304)**_** = −2.085**, ***p* = 0.038**^**t**^	1.2 (1.0)	1.5 (1.1)	**t**_**(304)**_** = −3.350**, ***p* = 0.001**^**t**^
Number of siblings^a^	1.7 (1.7)	1.9 (1.8)	t_(304)_ = −0.921, *p* = 0.358^t^	1.6 (1.7)	2.0 (1.8)	t_(304)_ = −1.661, *p* = 0.098^t^

N, number of patients; PwP, patients with polypharmacy; Pw/oP, patients without polypharmacy.

^a^Mean value (standard deviation); ^c^number of patients (%); ^Chi^Chi-square test; ^Fi^Fisher’s exact test; ^t^two-sample two-tailed Student’s t-test; *not assuming equal variances (Levene’s test: *p* < 0.05).

**Figure 1:**
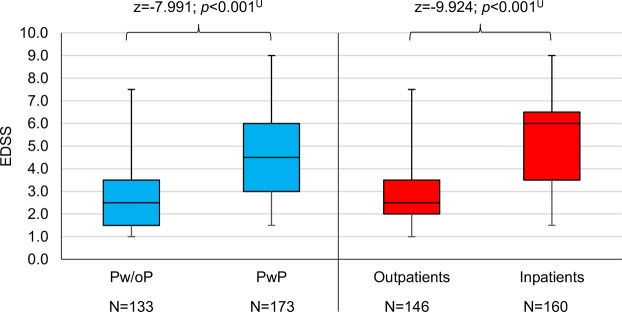
Comparison of the patients’ EDSS score with regard to polypharmacy and patient care. Comparing Pw/oP (N = 133) and PwP (N = 173) (according to all medications) as well as outpatients (N = 146) and inpatients (N = 160), the boxplot shows the distribution of the patients’ degree of disability for each subgroup. The upper and lower quartiles of the EDSS ratings are marked by the boxes. The whiskers range to the minimum and maximum values, while the medians are indicated by horizontal lines. EDSS, expanded disability status scale; N, number of patients; *p*, *p*-value; PwP, patients with polypharmacy; Pw/oP, patients without polypharmacy; ^U^Mann-Whitney U test.

**Figure 2:**
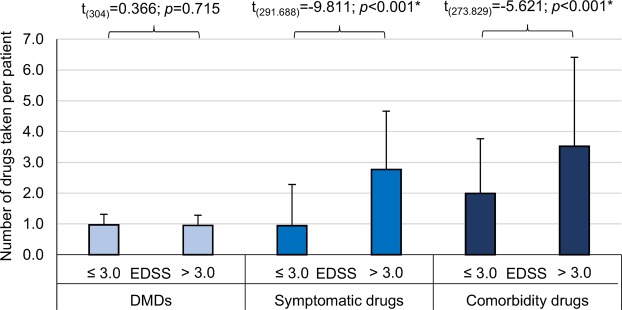
Number of drugs taken with respect to the patients’ degree of disability and therapy goals. The medications were split into three groups according to the therapy goals: DMDs, symptomatic drugs and comorbidity drugs. The patients were divided into two subgroups with EDSS ≤ 3.0 (N = 143) and >3.0 (N = 163), respectively. The bars mark the average number of drugs taken and the error bars show the standard deviation. Considering the drug intake of MS patients, the numbers of symptomatic and comorbidity drugs taken were significantly higher for patients with higher degrees of disability. DMDs, disease-modifying drugs; EDSS, expanded disability status scale; MS, multiple sclerosis; N, number of patients; t, two-sample two-tailed Student’s t-test; *not assuming equal variances (Levene’s test: *p* < 0.05).

**Figure 3:**
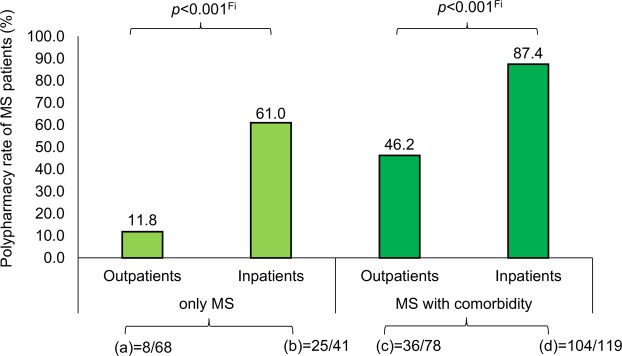
Polypharmacy rates regarding type of patient care and comorbidities. We split the patients (N = 306) into four groups according to the presence of comorbidities and inpatient/outpatient status. (**a**) Outpatients with MS only. (**b**) Inpatients with MS only. (**c**) Outpatients with MS and comorbidities. (**d**) Inpatients with MS and comorbidities. Regarding the polypharmacy rates of the four patient groups, MS inpatients with comorbidities showed the highest rate of polypharmacy. ^Fi^Fisher’s exact test; MS, multiple sclerosis.

The comparison of the clinical-neurological data between Pw/oP and PwP yielded significant differences for all investigated clinical factors (Table 4). As expected, PwP showed substantially higher disability levels than Pw/oP (Fig. 1) and had, on average, a longer disease duration. PwP were more frequently affected by SPMS, while Pw/oP predominantly had CIS/RRMS. All PPMS patients showed polypharmacy. It was apparent that approximately twice as many PwP had comorbidities compared to Pw/oP (Fisher’s exact test: *p* < 0.001). Furthermore, the proportion of inpatients with polypharmacy was two to three times higher than of those without polypharmacy (Fisher’s exact test: *p* < 0.001). These significant differences also remained following correction of the *p*-values for multiple testing (FDR < 0.05). Potential clinicodemographic factors predicting polypharmacy (considering all medications) were examined by using a multivariable logistic regression model with forward variable selection. This revealed that the presence of comorbidities (Wald_(1)_ = 26.620; *p* < 0.001; odds ratio [OR] = 4.879) and inpatient care (Wald_(1)_ = 25.253; *p* < 0.001; OR = 5.146) as well as higher EDSS scores (Wald_(1)_ = 11.769; *p* = 0.001; OR = 1.385) were significantly associated with the risk of polypharmacy (Figs 2 and 3).

**Table 4:** Clinical data comparison between patients with and without polypharmacy.

N	Polypharmacy (all medications)			Polypharmacy (long-term medications only)		
	Pw/oP	PwP	Statistics	Pw/oP	PwP	Statistics
	133	173		177	129	
EDSS^b^	2.5	4.5	**z = −7.991**, ***p* < 0.001**^**U**^	3.0	6.0	**z = −8.062**, ***p* < 0.001**^**U**^
Disease course^c^			**X**^**2**^_**(2)**_** = 74.871**, ***p* < 0.001**^**Chi**^			**X**^**2**^_**(2)**_** = 78.310**, ***p* < 0.001**^**Chi**^
CIS/RRMS	119 (89.5)	73 (42.2)		148 (83.6)	44 (34.1)	
SPMS	14 (10.5)	66 (38.2)		21 (11.9)	59 (45.7)	
PPMS	0 (0.0)	34 (19.7)		8 (4.5)	26 (20.2)	
Disease duration (Years)^b^	10	13	**z = −2.234**, ***p* = 0.025**^**U**^	9	15	**z = −4.592**, ***p* < 0.001**^**U**^
Comorbidities^c^			***p*** ** < 0.001** ^**Fi**^			***p*** ** < 0.001** ^**Fi**^
Pw/oSI	76 (57.1)	33 (19.1)		97 (54.8)	12 (9.3)	
PwSI	57 (42.9)	140 (80.9)		80 (45.2)	117 (90.7)	
Patient care^c^			***p*** ** < 0.001** ^**Fi**^			***p*** ** < 0.001** ^**Fi**^
Outpatients	102 (76.7)	44 (25.4)		116 (65.5)	30 (23.3)	
Inpatients	31 (23.3)	129 (74.6)		61 (34.5)	99 (76.7)	

CIS, clinically isolated syndrome; MS, multiple sclerosis; N, number of patients; PwP, patients with polypharmacy; PwSI, patients with secondary illnesses; Pw/oP, patients without polypharmacy; Pw/oSI, patients without secondary illnesses; PPMS, primary progressive MS; RRMS, relapsing-remitting MS; SPMS, secondary progressive MS.

^b^Median; ^c^number of patients (%) ^Chi^Chi-square test; ^Fi^Fisher’s exact test; ^U^Mann-Whitney U test.

Comparing the pharmacological data, the average number of medications taken was twice to three times as high in the PwP group than in the Pw/oP group (according to all medications: 8.1 vs. 2.6; according to long-term medications only: 8.8 vs. 3.4). Adding up the number of all medications taken by the 306 patients yielded a total of 1738 recorded medications (counted with repetitions). Despite the quite similar cohort sizes of PwP (N = 173) and Pw/oP (N = 133) in our study, 80.3% of the medications were taken by the PwP group.

The only medication categories without significant differences between Pw/oP and PwP were DMDs (z = −1.013; *p* = 0.311) when evaluating polypharmacy according to all medications and DMDs (z = −0.344; *p* = 0.731) and *pro re nata* (PRN) medications (z = −1.385; *p* = 0.166) when regarding long-term medications only (Table 5).

**Table 5:** Pharmacological data comparison between patients with and without polypharmacy.

N	Polypharmacy (all medications)			Polypharmacy (long-term medications only)		
	Pw/oP	PwP	Statistics	Pw/oP	PwP	Statistics
	133	173		177	129	
All medications^a^	2.6 (1.0)	8.1 (2.9)	**z = −15.075**, ***p* < 0.001**^**U**^	3.4 (1.8)	8.8 (2.9)	**z = −13.655**, ***p* < 0.001**^**U**^
Long-term medications^a^	2.0 (1.0)	6.4 (3.0)	**z = −13.040**, ***p* < 0.001**^**U**^	2.2 (1.1)	7.6 (2.5)	**z = −15.050**, ***p* < 0.001**^**U**^
PRN drugs^a^	0.6 (0.8)	1.7 (1.5)	**z = −7.154**, ***p* < 0.001**^**U**^	1.2 (1.5)	1.2 (1.2)	z = −1.385, *p* = 0.166^U^
Prescription-only drugs^a^	2.0 (1.0)	6.5 (2.9)	**z = −13.855**, ***p* < 0.001**^**U**^	2.6 (1.6)	7.1 (2.9)	**z = −12.859**, ***p* < 0.001**^**U**^
OTC drugs^a^	0.6 (0.7)	1.6 (1.5)	**z = −7.280**, ***p* < 0.001**^**U**^	0.8 (0.9)	1.7 (1.5)	**z = −6.367**, ***p* < 0.001**^**U**^
DMD^a^	0.9 (0.3)	1.0 (0.4)	z = −1.013, *p* = 0.311^U^	1.0 (0.4)	1.0 (0.3)	z = −0.344, *p* = 0.731^U^
Symptomatic drugs^a^	0.5 (0.7)	3.0 (1.8)	**z = −11.990**, ***p* < 0.001**^**U**^	1.0 (1.3)	3.2 (1.9)	**z = −10.301**, ***p* < 0.001**^**U**^
Comorbidity drugs^a^	1.1 (0.9)	4.1 (2.6)	**z = −11.561**, ***p* < 0.001**^**U**^	1.5 (1.2)	4.7 (2.7)	**z = −11.365**, ***p* < 0.001**^**U**^

DMD, disease-modifying drugs; N, number of patients; OTC, over-the-counter; PRN, *pro re nata*; PwP, patients with polypharmacy; Pw/oP, patients without polypharmacy.

^a^Mean value (standard deviation) of the number of drugs taken per patient; ^U^Mann-Whitney U test.

In terms of routes of drug administration, PwP took twice to over three times more intravenously, perorally and subcutaneously administered drugs than Pw/oP (Table 6). These significant differences remained after correcting the *p*-values for multiple testing (FDR < 0.05).

**Table 6:** Comparison of routes of drug administration with respect to the presence of polypharmacy.

Route of administration	Polypharmacy (all medications)			Polypharmacy (long-term medications only)		
	Pw/oP	PwP	Statistics	Pw/oP	PwP	Statistics
N	133	173		177	129	
buccal^**a**^	0.0 (0.0)	0.0 (0.2)	z = −1.762, *p* = 0.078^U^	0.0 (0.2)	0.0 (0.2)	z = −1.327, *p* = 0.185^U^
conjunktival^**a**^	0.0 (0.0)	0.1 (0.3)	**z = −2.957**, ***p* = 0.003**^**U**^	0.0 (0.1)	0.1 (0.3)	**z = −3.333**, ***p* = 0.001**^**U**^
cutaneous^**a**^	0.0 (0.1)	0.1 (0.3)	**z = −2.340**, ***p* = 0.019**^**U**^	0.0 (0.1)	0.1 (0.3)	**z = −2.712**, ***p* = 0.007**^**U**^
intramuscular^**a**^	0.1 (0.2)	0.0 (0.2)	z = −0.757, *p* = 0.449^U^	0.1 (0.3)	0.0 (0.2)	z = −0.856, *p* = 0.392^U^
intravenous^**a**^	0.4 (0.5)	0.8 (0.7)	**z = −5.141**, ***p* < 0.001**^**U**^	0.5 (0.6)	0.8 (0.6)	**z = −3.868**, ***p* < 0.001**^**U**^
nasal^**a**^	0.0 (0.1)	0.0 (0.1)	z = −0.187, *p* = 0.852^U^	0.0 (0.1)	0.0 (0.1)	z = −0.225, *p* = 0.822^U^
peroral^**a**^	1.7 (1.0)	6.2 (2.6)	**z = −14.381**, ***p* < 0.001**^**U**^	2.3 (1.5)	6.9 (2.6)	**z = −13.596**, ***p* < 0.001**^**U**^
pulmonary^**a**^	0.0 (0.1)	0.1 (0.2)	z = −1.307, *p* = 0.191^U^	0.0 (0.1)	0.1 (0.3)	**z = −2.197**, ***p* = 0.028**^**U**^
rectal^**a**^	0.0 (0.0)	0.0 (0.2)	z = −1.242, *p* = 0.214^U^	0.0 (0.2)	0.0 (0.1)	z = −0.220, *p* = 0.826^U^
subcutaneous^**a**^	0.4 (0.6)	0.8 (0.7)	**z = −5.332**, ***p* < 0.001**^**U**^	0.5 (0.6)	0.8 (0.7)	**z = −4.865**, ***p* < 0.001**^**U**^
sublingual^**a**^	0.0 (0.1)	0.0 (0.2)	z = −0.803, *p* = 0.422^U^	0.0 (0.1)	0.0 (0.2)	z = −0.038, *p* = 0.970^U^
vaginal^**a**^	0.0 (0.0)	0.0 (0.2)	z = −1.762, *p* = 0.078^U^	0.0 (0.1)	0.0 (0.1)	z = −0.319, *p* = 0.750^U^

N, number of patients; PwP, patients with polypharmacy; Pw/oP, patients without polypharmacy.

^a^Mean value (standard deviation) of the number of drugs taken per patient; ^U^Mann-Whitney U test.

**Figure 4:**
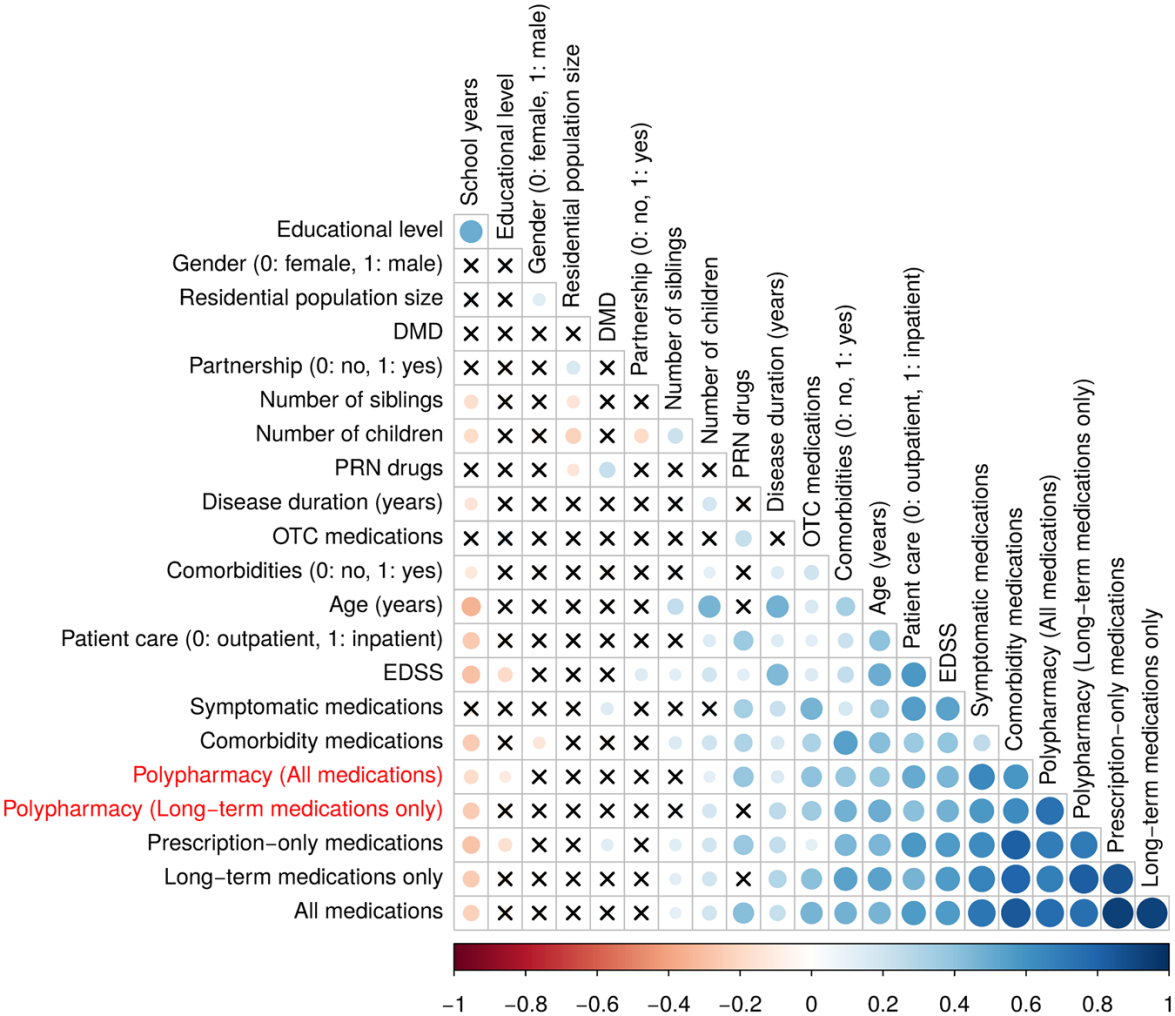
Correlation matrix visualization of the correlation between variables and polypharmacy status. Using the “corrplot” R package, the correlation matrix heatmap was built. The degree of pairwise correlation with respect to Pearson’s correlation coefficient is displayed by the colour gradient. The crosses represent absence of significance (*p*-values > 0.05). A significant correlation was seen, for instance, between age and comorbidities. DMD, disease-modifying drug; EDSS, expanded disability status scale; OTC, over-the-counter; PRN, *pro re nata*.

Pearson’s correlation coefficients demonstrated significant relations between several variables, as illustrated in the correlation matrix in Fig. 4. For instance, EDSS and age correlated significantly with each other.

### Drug spectrum

**Figure 5:**
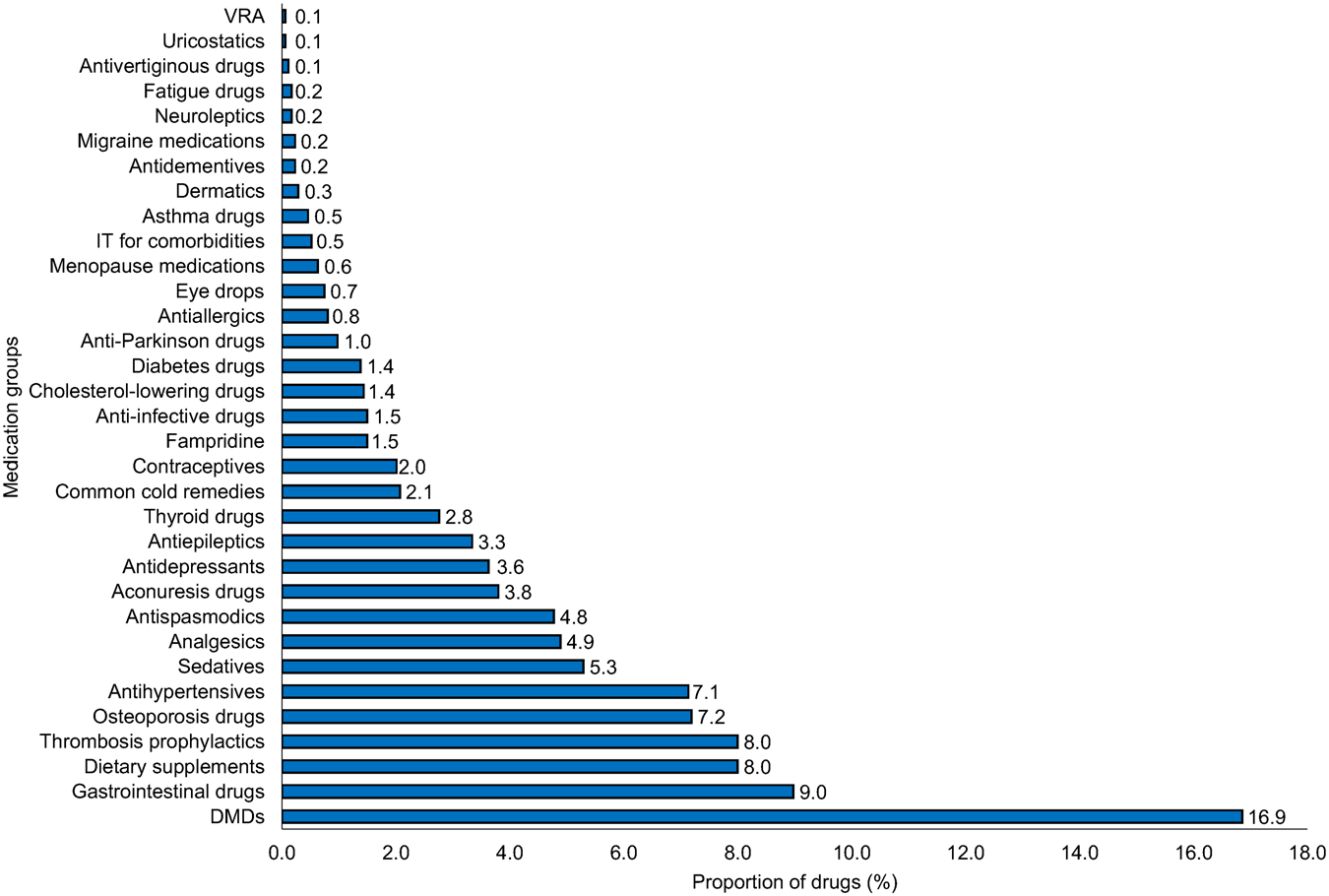
Proportion of categories of medications used by MS patients. The proportions of the medication groups were calculated according to the total number of drugs taken by the 306 patients included in our study (N = 1738). The proportions ranged from 0.1% (VRA, uricostatics) to 16.9% (DMDs). DMDs, disease-modifying drugs; IT, immunotherapy; MS, multiple sclerosis; N, number of medications; VRA, vasopressin receptor antagonists.

The most frequently used medication groups in our study were DMDs (16.9%), gastrointestinal drugs (9.0%), dietary supplements (8.0%), thrombosis prophylactics (8.0%), osteoporosis medications (7.2%) as well as antihypertensives (7.1%) (Fig. 5).

Significant differences between PwP and Pw/oP in the frequency of drugs taken emerged for all of the aforementioned medication groups except for the DMDs (Table 7). On average, PwP, older patients and inpatients took drugs more frequently than Pw/oP, younger patients and outpatients. As an exception, contraceptives were taken more often by Pw/oP, outpatients and younger patients. There were no significant differences for antiallergics, anti-dementia drugs, dermatics, fatigue medications, menopause medications, migraine medications, neuroleptics, thyroid medications, uricostatics and vasopressin receptor antagonists (Fisher’s exact test: *p* > 0.05).

**Table 7:** Comparison of medications regarding polypharmacy, age and patient care.

N	Polypharmacy (all medications)			Polypharmacy (long-term medications only)			Age (Years)			Patient care		
	Pw/oP^a^	PwP^a^	FDR^Fi^	Pw/oP^a^	PwP^a^	FDR^Fi^	<60^a^	≥60^a^	FDR^Fi^	OP^a^	IP^a^	FDR^Fi^
	133	173		177	129		247	59		146	160	
Aconuresis drugs	8.3	26.6	** <0.001**	7.3	34.1	** <0.001**	17.0	25.4	0.243	9.6	26.9	** <0.001**
Analgesics	18.0	31.8	**0.015**	23.2	29.5	0.298	25.5	27.1	1.000	25.3	26.3	1.000
Antiallergics	3.8	4.6	0.956	4.5	3.9	1.000	5.3	0.0	0.167	5.5	3.1	0.730
Antidementives	0.0	2.3	0.194	0.0	3.1	0.051	0.8	3.4	0.266	0.7	1.9	0.907
Anti-depressants	8.3	26.6	** <0.001**	9.0	31.8	** <0.001**	15.8	30.5	0.055	12.3	24.4	**0.024**
Antiepileptics	3.0	27.7	** <0.001**	4.0	34.9	** <0.001**	13.0	33.9	** <0.001**	7.5	25.6	** <0.001**
Antihyper-tensives	6.0	39.9	** <0.001**	8.5	48.1	** <0.001**	17.8	55.9	** <0.001**	11.6	37.5	** <0.001**
Anti-infective drugs	0.8	12.7	** <0.001**	1.7	15.5	** < 0.001**	5.7	15.3	0.076	4.1	10.6	0.108
Anti-Parkinson drugs	0.8	7.5	**0.010**	1.7	8.5	**0.019**	3.6	8.5	0.257	2.1	6.9	0.113
Antispas-modics	3.8	35.8	** <0.001**	9.0	39.5	** <0.001**	19.4	32.2	0.094	6.8	35.6	** <0.001**
Antivertiginous drugs	0.0	1.2	0.669	0.0	1.6	0.243	0.0	3.4	0.094	0.0	1.3	0.823
Asthma drugs	0.0	3.5	0.063	0.0	4.7	**0.010**	1.2	5.1	0.171	2.1	1.9	1.000
Cholesterol-lowering drugs	0.8	13.9	** <0.001**	1.1	17.8	** <0.001**	4.0	25.4	** <0.001**	2.7	13.1	**0.003**
Common cold remedies	5.3	15.0	**0.015**	8.5	14.0	0.199	9.3	16.9	0.189	11.0	10.6	1.000
Contraceptives	15.0	8.7	0.162	16.4	4.7	**0.004**	14.2	0.0	** <0.001**	19.9	3.8	** <0.001**
Dermatics	1.5	1.7	1.000	1.1	2.3	0.716	1.6	1.7	1.000	1.4	1.9	1.000
Diabetes drugs	0.8	8.1	**0.007**	2.3	8.5	**0.028**	3.6	10.2	0.106	2.1	7.5	0.080
Dietary supplements	21.1	38.7	**0.003**	23.7	41.1	**0.004**	31.2	30.5	1.000	32.2	30.0	0.979
DMDs	92.5	91.9	1.000	91.5	93.0	0.716	92.3	91.5	0.967	95.9	88.8	0.079
Eye drops	0.0	6.4	**0.007**	0.6	7.8	**0.003**	2.4	8.5	0.097	2.7	4.4	0.858
Fampridine	0.8	14.5	** <0.001**	5.1	13.2	**0.036**	7.7	11.9	0.455	4.1	12.5	**0.033**
Fatigue drugs	0.0	1.7	0.358	0.0	2.3	0.111	0.8	1.7	0.653	0.7	1.3	1.000
Gastrointes-tinal drugs	11.3	68.2	** <0.001**	24.9	69.0	** <0.001**	37.2	69.5	** <0.001**	11.6	72.5	** <0.001**
IT for comorbidities	0.0	4.0	**0.035**	0.0	5.4	**0.004**	1.2	6.8	0.084	1.4	3.1	0.783
Menopause medications	1.5	5.2	0.183	1.7	6.2	0.091	4.0	1.7	0.885	2.1	5.0	0.435
Migraine medications	0.8	1.2	1.000	1.1	0.8	1.000	1.2	0.0	1.000	0.7	1.3	1.000
Neuroleptics	0.8	1.2	1.000	0.6	1.6	0.654	0.8	1.7	0.653	0.7	1.3	1.000
Osteoporosis drugs	17.3	49.1	** <0.001**	22.0	53.5	** <0.001**	30.8	54.2	**0.004**	22.6	46.9	** <0.001**
Sedatives	8.3	44.5	** <0.001**	18.1	43.4	** <0.001**	24.3	47.5	**0.004**	4.8	50.6	** <0.001**
Thrombosis prophylactics	7.5	64.7	** <0.001**	18.6	69.0	** <0.001**	31.6	74.6	** <0.001**	7.5	69.4	** <0.001**
Thyroid drugs	13.5	15.6	0.800	12.4	17.8	0.257	14.2	16.9	0.777	15.8	13.8	0.907
Uricostatics	0.0	0.6	1.000	0.0	0.8	0.497	0.4	0.0	1.000	0.0	0.6	1.000
VRA	0.0	0.6	1.000	0.0	0.8	0.497	0.4	0.0	1.000	0.0	0.6	1.000

DMD, disease-modifying drug; FDR, adjusted *p*-value according to false discovery rate; IP, inpatients; IT, immunotherapy; OP, outpatients; PwP, patients with polypharmacy; Pw/oP, patients without polypharmacy; VRA, vasopressin receptor antagonists.

^a^Frequency of use of medication groups (%); ^Fi^Fisher’s exact test.

## Discussion

Our prospective single-center study aimed to examine the prevalence of polypharmacy in a cohort of MS patients as well as possible influencing factors associated with polypharmacy. To date, there are only few studies addressing polypharmacy in MS patients^37–40^. The available studies analyzed quality of life and relapse rate^38^, occurring symptoms such as fatigue and cognitive ability^37^, the use of antiepileptic drugs or antidepressants^39^ and the aspect of rehospitalization^40^. The focus of our study was on presenting a real-world snapshot of a German MS cohort with respect to the prevalence of polypharmacy, supplemented by an investigation of the associations of polypharmacy with sociodemographic and clinical-neurological factors. Moreover, we considered the whole range of medications, identifying the most frequent medication groups used by the examined MS patients and in particular by PwP.

The study population showed an average age of 48.7 years, resembling values found in other studies examining polypharmacy in MS^37–39^. A low employment rate (37.6% in our study) despite the relatively young average age can be explained by the limiting nature of the disease, e.g. due to fatigue and cognitive disturbances^15,41^. Even at lower levels of disability, such impairments can lead to an incapacity to work.

As expected, the patients’ degree of disability laid in the moderate range with a median EDSS score of 3.5. For the German MS registry (N = 48386 patients in the registry population), a median EDSS score of 3.0 was reported^42^. Similar findings were described in other studies^37,42^. The MS subtype proportions in our study (62.7% CIS/RRMS, 26.1% SPMS and 11.1% PPMS) were in accordance with the expected distribution^43^. With regard to hospitalization, there were approximately equal numbers of outpatients and inpatients in our study. The treatment of SPMS and PPMS with, for instance, glucocorticosteroid (GCS) pulses or triamcinolone acetonide took place in the inpatient setting^44,45^. For this reason, inpatients included more SPMS and PPMS patients, while CIS/RRMS patients more frequently received outpatient care.

There are various ways to define polypharmacy: The division into minor polypharmacy (two to four medications) and major polypharmacy (five or more medications)^46^, the prescription of two or more medications with the same therapeutic objective^47,48^ or two or more medications which belong to the same chemical substance class^47^. However, the most common definition of polypharmacy is exceeding a certain number of medications^29^. In our study, polypharmacy was defined as the use of five or more medications, as this definition is well-established and frequently used in the literature^20–25^.

The proportion of PwP in the present study was 56.5% when analyzing polypharmacy according to all medications. The second classification, in which *pro re nata* drugs (PRN) were excluded, yielded a polypharmacy rate of 42.2%. These polypharmacy rates of our MS cohort resemble those of other polypharmacy studies on MS patients, reporting rates of 14.9%^38^ to 59%^39^. The rate of 14.9% was relatively low because first- and second-generation DMDs, general immunosuppressants and GCS^38^ have not been considered for examining polypharmacy.

Distinguishing polypharmacy by including or excluding PRN drugs offers, on the one hand, the opportunity to take a general view on all medications and, on the other hand, the investigation of medications which are taken regularly and on a long-term basis. Comparing these two definitions, the analysis considering all medications may provide a broader assessment because many patients additionally take as-needed medications like OTC and herbal preparations^38^.

Regarding sociodemographic data, the relatively high average age and high retirement rate in the group of PwP can be attributed to the increasing likelihood of suffering from comorbidities with age. Earlier studies have already demonstrated that a higher age at the time of MS diagnosis is associated with comorbidities^49,50^. Accordingly, the number of medications taken also rises with age.

The association between higher EDSS scores and polypharmacy is paralleled by higher proportions of SPMS and PPMS patients among PwP^51^ and, consequently, a higher proportion of inpatients in the PwP group. Moreover, the significantly higher age of the PwP explains the significantly longer mean disease duration compared to Pw/oP^37^. A significant difference between Pw/oP and PwP also emerged in terms of comorbidities: Among the PwP, comorbidities were almost twice as prevalent as among the Pw/oP. There are two major reasons for this observation: First, the occurrence of comorbidities leads to an increasing number of medications taken. Secondly, certain MS drugs can cause secondary illnesses and side effects^52^, requiring further medical interventions^12^.

Generally, distinguishing between comorbidities as separate diagnoses and disease symptoms is a debated issue. For instance, is depression a comorbidity or a symptom of MS? In some studies, depression has been associated with more lesions at particular brain areas and so it could be a secondary manifestation of MS^53^. However, there is no consistent causality. Consequently, for implementing a more general definition of comorbidities, we followed the principles laid down by the “International Workshop on Comorbidities in MS”^54–62^.

The more detailed analysis of the pharmacological data revealed that PwP took, on average, much more drugs than Pw/oP (mean values: 8.1 vs. 2.6). The DMDs did not contribute to this quantitative medication difference between Pw/oP and PwP (Table 5), as immunotherapy in MS is generally maintained as a monotherapy^63^. Accordingly, a higher number of DMDs among PwP was not to be expected. Twenty-three (7.5%) of the 306 patients were not currently taking any DMD. Some of these patients were in the process of having their treatment adjusted or opted to stop the treatment due to side effects^12^. Nine patients (2.9%) had two DMDs in their medication plans, which have been recorded in the patient interview and by reviewing the patient records. In each case, one of these two medications was a GCS which was used to treat an acute relapse occurring at the time of the survey. All other 274 patients (89.5%) have taken precisely one DMD. Following the guidelines of the German Neurological Society, an early initiation of DMD treatment is recommended after diagnosis. Thus, few MS patients are not treated. Recent data of a German National MS Cohort showed that after a median time of 167 days, the majority of early-stage CIS/RRMS patients (762/1124) started DMD therapy^64^. In our study, the median disease duration was 11 years, so nearly all patients used DMDs.

Regarding the routes of drug administration, peroral medications constituted the largest share of routes of administration in this study, with 74.1%. The finding that the majority of the recorded medications were administered in this way, which is generally the most popular one^65^, can be explained by the fact that peroral administration is easy to understand, uncomplicated and well-manageable.

Examining the question of which sociodemographic and clinical-neurological factors in combination are substantially associated with polypharmacy, the following results emerged: On the one hand, polypharmacy was correlated with higher levels of disability (Wald_(1)_ = 11.769; *p* = 0.001; OR = 1.385). Thus, with each 1.0 step on the EDSS, the risk of polypharmacy rises by 38.5%. This may be referred to the increase of medical therapies, which are used to compensate and to reduce symptoms like spasticity^14^, pain^16^ and gait disturbances^66^. Furthermore, the presence of secondary illnesses was associated with polypharmacy (Wald_(1)_ = 26.620; *p* < 0.001; OR = 4.879). Thus, the risk of polypharmacy among patients with secondary illnesses (PwSI) was almost five times as high as in patients without secondary illnesses (Pw/oSI), which can be attributed to the additionally prescribed treatments due to the occurrence of comorbidities. There was also an association between the type of patient care and polypharmacy: Inpatients had a five times higher risk of polypharmacy than outpatients (Wald_(1)_ = 25.253; *p* < 0.001; OR = 5.146). An explanation for this may be that the inpatients in our study were mostly SPMS or PPMS patients who generally show higher EDSS scores than RRMS patients^51^. More strongly disabled patients were therefore mostly treated in the inpatient setting. Moreover, inpatients are more likely taking prophylactic medications, e.g. thrombosis prophylactics, leading to a further rise in the number of medications taken.

However, the patient care differs among countries. For instance, Germany and France have a distinctive inpatient healthcare system, while patients in the United Kingdom and Canada are mostly treated in outpatient settings^67,68^. There are many global differences in the access and management of MS treatment^69^. In some countries, governments and health insurances do not fully compensate for the costs of DMD treatment^1,70,71^. Access to DMDs is strongly dependent on treatment costs. For a US MS patient, the costs of DMD treatment (at least US$ 50,000 per year) are two to three times higher than in Australia or Canada^72^. Despite these differences, the essential associations found in our study should be generalizable for other countries.

In the analysis of the spectrum of medications, DMDs, osteoporosis medications, antihypertensives, gastrointestinal drugs, thrombosis prophylactics and dietary supplements were identified as the most frequently used medication groups in MS in our study. DMDs form the basis for MS immunotherapy to prevent relapses and to alleviate the progression of the disease^2^. There is evidence that patients with cardiovascular diseases are at higher risk of developing a further one^73^ and so those patients are more likely to use more than one cardiovascular drug. This matter as well as higher age and therapeutic side effects are related to the observed more frequent use of antihypertensives^74^, gastrointestinal drugs^75^ and osteoporosis drugs^76^. The majority of thrombosis prophylactics and proton pump inhibitors in our study were administered during the inpatient hospital stay for GCS pulse treatment, following the German guidelines for the diagnosis and treatment of MS^77^. The use of dietary and herbal supplements in the general population has become a trend^78,79^ because they are available in any price range and prescriptions by physicians are not necessary. Dietary supplements may be useful to support MS treatment. However, further studies are necessary to evaluate the effect of supplements on relapse rate and progression of MS^80,81^.

The establishment of a well-thought-out medication management considering an adjustment of medications is essential to optimize treatment. The patients’ medication plans should be regularly checked by physicians and pharmacists for identifying unnecessary or missing prescriptions as well as drug interactions. Therefore, a well working network between the patients’ physicians and pharmacists has to be established. Furthermore, evidence-based, non-medicinal approaches such as physiotherapy^82–84^ or cognitive-behavioral talking therapy^85^ can offer alternatives to or complement medications. In addition, there is evidence that the mortality risk of older polypharmacy patients can be reduced by a healthy lifestyle^86^.

Limitations of this study that should be mentioned are the lack of controls and the absent explanatory power regarding causality. A longitudinal study with controls could reveal new insights on causal relationships and could point out further MS-specific factors underlying polypharmacy. Nonetheless, this study gave a current overview of the prevalence and the medication situation in a large representative single-center MS cohort. Our study may be the starting point for further studies addressing testable hypotheses.

In summary, our study showed that polypharmacy plays an important role for MS patients. Polypharmacy in MS is linked to higher degrees of disability, the presence of comorbidities and an inpatient treatment scenario. Future computational analyses of the medication plans of MS patients could be conducted to assess potential and clinically relevant medication interactions.

## Materials and Methods

This cross-sectional study was conducted at the Department of Neurology and the Division of Neuroimmunology at the University Medicine Rostock. The data were collected between March 2017 and May 2018. In order to gather sociodemographic data, clinical-neurological data and current medication data, patients were examined by the following means: anamnesis, structured patient interview, patient records and clinical examination. The study was conducted in accordance with the EU General Data Protection Regulation. The inclusion criterion for this study was a confirmed diagnosis of MS or CIS according to the revised McDonald criteria from 2010^87^. Overall, 309 patients attended the examination. Three of them declined to participate in the study for personal reasons. Thus, 306 patients were included and analyzed in this study. Prior informed consent was obtained from all individual participants included in the study. This study was approved by the University of Rostock’s ethics committee (permit number A 2014-0089) and carried out in line with the Declaration of Helsinki.

### Data collection

The patients were examined with respect to sociodemographic, clinical-neurological and pharmacological factors.

Sociodemographic data: These included gender, age, number of school years (not including time spent in training or higher education), educational level, employment status and place of residence, with the latter subdivided into rural community (<5000 residents), small town (5000–20000 residents), medium-sized town (20000–100000) and city (>100000). Moreover, partnership status, number of children and number of siblings were recorded.

Clinical-neurological data: To categorize the degree of disability, Kurtzke’s Expanded Disability Status Scale (EDSS) was used^88^. MS subtypes were classified into RRMS, PPMS, SPMS and patients with CIS (N = 6)^89^. Furthermore, we recorded the disease duration since the time of the initial diagnosis of MS/CIS as well as data on the presence of comorbidities (Pw/oSI and PwSI) and the type of patient care (outpatient, inpatient).

Pharmacological data: The trade name, indication, active ingredients, dosage and route of administration were obtained for the various preparations from the patients’ medication plans.

To ensure the completeness of the collected data, for each patient, a structured patient interview and a review of the medical records were conducted. In the analysis, we only considered medications which were actually taken as stated by the patients.

### Inpatient and outpatient scenario

At the Department of Neurology of the University of Rostock, there are wards for inpatients as well as for outpatients. At the outpatients’ ward, patients with a usually stable disease situation had a routine appointment. While waiting for the checkup there, the outpatients were asked to participate in this study. After patients’ agreement, the structured interview and the review of the medical records were performed. At the inpatients’ ward, there were patients with more severe disease courses, patients with an increase in disease activity and patients with adverse events during DMD therapy. Usually, inpatients staid there a couple of days. During this time, the patients were asked to take part in this study and after their agreement, the interview and the patient record review were conducted.

### Medication analysis

A more precise analysis of the medications was undertaken by evaluating them according to three criteria.

Dosing schedule: A distinction was made between long-term and as-needed (*pro re nata*; PRN) medications. Long-term medications are those taken daily or at regular intervals, e.g. once a week or once a month. Such medications are used to treat long-term illnesses or complaints. In contrast, PRN medications are taken at irregular intervals to treat acute or sporadic complaints.

Prescription status: A distinction was made between prescription-only and OTC preparations.

Therapeutic objective: A distinction was made between DMDs^90^, specific symptomatic drugs for MS and medications to treat comorbidities. The symptomatic drugs aim at a targeted alleviation of specific MS symptoms, such as spasticity or pain. Medications to treat comorbidities comprise drugs for the therapy of secondary illnesses and for other reasons, e.g. contraception.

### Definition of polypharmacy and comorbidities

The number of five medications was set as the threshold to compare Pw/oP and PwP. Accordingly, patients who took five or more medications were counted as PwP. This constitutes the most common definition of polypharmacy^20–25^. Polypharmacy was analyzed, on the one hand, according to the total number of medications (i.e. the sum of long-term and PRN medications) and, on the other hand, according to the number of long-term medications only.

For defining comorbidities, we followed the study by Laroni *et al*.^49^ and adhered to the recommendations for observational studies of comorbidity in MS by Marrie *et al*.^55^ (“International Workshop on Comorbidities in MS”)^54,56–62^. Accordingly, PwSI were defined based on the patient records and the physicians’ expert opinion. Patients without comorbidities were categorized as Pw/oSI, while those categorized as PwSI had to have at least one comorbidity.

### Statistical analysis

Statistical analyses were conducted using PASW Statistics 18 (IBM). Patient data were anonymized before entry into the database. The collected data were tested for homogeneity of variances (Levene’s test). For the comparative analysis of Pw/oP and PwP, we used two-sample two-tailed Student’s t-tests, Fisher’s exact tests, Chi-square tests and Mann-Whitney U tests. To determine which associations between polypharmacy (defined by the total number of medications taken) as response variable and eight sociodemographic variables (gender, age, number of school years, educational level, partnership, place of residence, number of children and number of siblings) as well as four clinical-neurological variables (comorbidities, inpatient/outpatient care, disease duration and EDSS) as explanatory variables could be seen as statistically significant, we conducted a stepwise binary logistic regression with forward variable selection based on the likelihood ratio. The significance level was set at α = 0.05. To take into account alpha error inflation in the case of multiple testing, the *p*-values were corrected according to FDR^91^. The pairwise interdependencies between various variables were identified by the analysis of Pearson’s correlation coefficients. Using the “corrplot” R package, we obtained a correlation matrix.

## Data Availability

The datasets generated and analyzed in the current study are available from the corresponding author on reasonable request.
